# Climate change risk perception in the USA and alignment with sustainable travel behaviours

**DOI:** 10.1371/journal.pone.0244545

**Published:** 2021-02-03

**Authors:** Jean Fletcher, James Higham, Nancy Longnecker

**Affiliations:** 1 Department of Science Communication, University of Otago, Dunedin, New Zealand; 2 Department of Tourism, University of Otago, Dunedin, New Zealand; 3 Norwegian School of Hotel Management, University of Stavanger, Stavanger, Norway; Universitat Autonoma de Barcelona, SPAIN

## Abstract

In an online survey of 1071 Americans conducted in October 2016, we found technological optimism, environmental beliefs, and gender to be better predictors of climate change concern than respondents’ perceived ability to visualize the year 2050 and their future optimism. An important finding from this study is that in October 2016, just before the 2016 Presidential election, 74% of responding Americans were concerned about climate change. Climate change ranked as their second most serious global threat (behind terrorism). However, when asked to describe travel in the year 2050 only 29% of participants discussed lower carbon options, suggesting that actively envisioning a sustainable future was less prevalent than climate change concern. Enabling expectations and active anticipation of a low carbon future may help facilitate mitigation efforts.

## Introduction

The Intergovernmental Panel on Climate Change (IPCC) was created in 1988 to summarize climate change research and to provide realistic response recommendations (http://www.ipcc.ch/). More than thirty years later, climate change continues to be a monumental problem [1]. The 2015 Paris Agreement was created as an international action plan to address climate change and was heralded as a political triumph for climate change mitigation [2]. Unfortunately, the United States announced its withdrawal from the Paris Agreement on June 1^st^, 2017 [3]. This is problematic as the U.S. is a significant emitter of greenhouse gases [3, 4]. In terms of the global carbon footprint (CF) of travel and tourism, the USA ranks first [5]. Therefore, successful mitigation of climate change at the global level will require American participation.

Understanding how Americans judge climate change risk is important because people’s concern about climate change may influence their behaviours [6] or behavioural intentions [7]. There is a growing body of literature exploring how psychological distance may influence perceptions of climate change risk [8–12]. Psychological distance refers to an individual’s perception of how close something (for example a person, event, or time) is to their self [13]. It has been proposed that time, physical space, uncertainty and social identity can contribute to how psychologically distant people feel from climate change and its repercussions [8–13]. Our study adds to this discussion by exploring if temporal distance, specifically the notion that climate change may be perceived to involve distant future events, affects how Americans’ judge climate change risk. We hypothesized that participants who find it difficult to visualize the distant future and who are more optimistic about the future would be less concerned about climate change.

Our study was conducted at an interesting time in American climate change history. We surveyed Americans in late October 2016, almost two months after the United States officially joined the Paris Agreement [14] and three weeks before climate change denialist Donald Trump was elected as American president. Our study provides insight into the American public’s perception of climate change risk at this pivotal time in American and world history. We should also note that our survey was conducted three years before the Covid-19 pandemic drastically affected global travel.

There are three sections to this paper. The first part lays out a theoretical framework proposing that a person’s ability to visualize the distant future (represented as the year 2050) and their optimism regarding the future influences how they judge climate change risk. The second part of the paper proceeds to test our proposed framework and considers whether other factors may be better predictors of climate change risk perception. The third section explores what participants envision the future (specifically travel in the year 2050) looking like and whether climate mitigating options are depicted in their descriptions.

### Theoretical framework

The IPCC has often discussed climate change in reference to the years 2050 or 2100 [1]. This could give people the impression that climate change is a future problem instead of a current problem. Whether people think about climate change as a current or future problem is important. Trope and Liberman’s Construal Level Theory (CLT) argues that a person thinks about an event differently if they expect it to happen tomorrow as opposed to some point in the medium to long-term future [15]. According to this theory, when climate change is perceived to be a temporally distant threat it will be approached differently than when it is perceived to be an immediate threat [13]. Brügger, Morton, and Dessai explained that this is because individuals will utilize different information when making proximal versus distant climate change decisions [16]. For example, their experiments on university students in the U.K. found that for participants who were asked questions in the context of climate change in their area (the U.K.), fear was a stronger factor in determining their climate change decisions. In contrast, for participants who were asked questions in the context of the wider world, scepticism was a stronger factor in their same climate change decisions [16].

Studies from Australia [8], United Kingdom [9], and the United States [10] have reported correlations between when participants expect climate change to affect an area and their level of concern about climate change. Interestingly, research from Israel found that because it was perceived to be a distant threat, climate change was considered to be less threatening to participants, regardless of how much concern it generated [17]. According to the CLT, the fact that people prioritize threats they perceive to be more imminent over those they consider distant even if the distant threat is believed to be more severe suggests that when making risk assessments, perceived temporal distances influences how much weight an individual gives to the severity of an event when making their decision.

**Fig 1:**
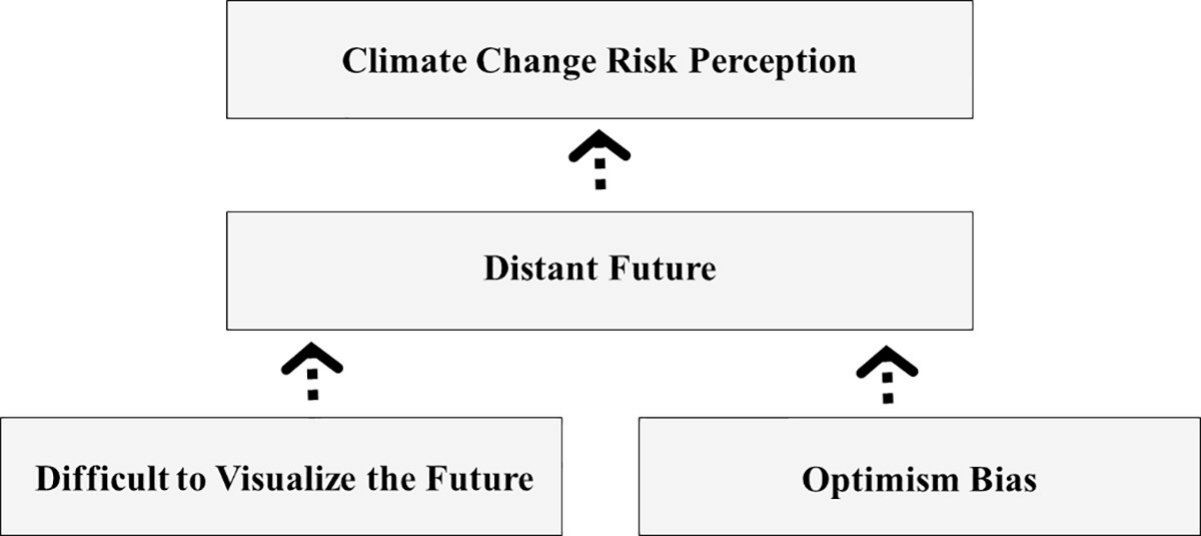
Theoretical framework that guided this research.

Unlike previous studies, our study explores temporal distance by focusing on two future related factors (Fig 1). In the following sections we discuss how thinking about the distant future in an abstract and unclear way may make it difficult to assess the implications of climate change. We also consider how optimism about the future, particularly regarding the ability of technology to be able to mitigate the impact of climate change, could result in people underestimating the likelihood of the consequences of climate change. Optimism bias occurs when an individual believes their future will be better than that of their peers even though statistically and realistically it probably won’t be [18]. By focusing on participants’ abilities to visualize the distant future and their future optimism, we aim to better understand what information may be used when making climate change risk judgements.

#### Difficulty visualizing future consequences of climate change

When judging climate change risk, it is important to consider the potential consequences of climate change, but since these consequences are often reported in the timeframes of 2050 or 2100 [1], assessing climate change risk realistically requires the ability to imagine a world more than 25 years into the future. The problem is that people have difficulty visualizing that far ahead [19]. This may make it difficult for people to visualize the consequences of both mitigation and inaction. In fact, the further into the future people imagine, the longer it takes them to visualize [20] and the less clear the visualisation becomes [19, 20], a point no doubt influenced by the acceleration of technologies and the rapid pace of change in contemporary societies. This process can be further affected by age [21] and habitual emotional suppression [22].

The abstract nature of the future may inspire procrastination as people wait for more information before making a decision [15]. This has important implications for climate change as the cumulative nature of carbon emissions means the longer it takes to reduce emissions, the more drastic the emissions reduction needs to be. While the near future is fairly concrete and contextualized the distant future is more abstract [15]. Because of this, people may have a difficult time visualizing the consequences of how climate change will affect their lives in the distant future. This may inhibit their ability to assess the risk of climate change, as well as their willingness to engage in mitigation. Sheppard et al. found that Canadians were more motivated to change their behaviours when they were presented with computer-generated pictures of how climate change might affect their local community which helped them to visualize these consequences [23].

#### Optimism bias

Another way in which the futuristic nature of climate change might influence peoples’ perception of climate change risk involves optimism. People are more likely to imagine positive futures compared to negative futures [24]. Therefore, people may have trouble imagining climate change as a *personally* negative thing. When university students were asked to describe their futures, Newby-Clark and Ross found that participants discussed mostly positive experiences [24]. However, students discussed both positive and negative experiences when talking about the past. Researchers followed up this experiment by specifically asking students to imagine both positive and negative futures and found that it took participants longer to imagine negative futures than it did positive [24]. Students were more likely to believe positive futures would occur compared to the negative futures [24]. Similarly, undergraduates were more likely to be optimistic about their personal futures compared to the world’s future [25]. Optimism has been seen in about 80% of people regardless of race, religion and age [18].

When applied to climate change, personal optimism bias may make people believe that the effects of climate change will not be that severe or that climate change will not affect them. A 2019 study found that although 67% of Americans agreed that climate change was already occurring, 42% believed it would have little to no effect on themselves personally [26]. This optimistic perception that an individual’s future will turn out positively, despite any statistics or logic that would suggest otherwise, may make people less concerned and less willing to engage in climate change mitigation. However, tackling optimism bias is fraught as a lack of optimism bias is linked with depression [18]. As discussed by Harré, there is a delicate balance between making people concerned enough about their own future to act but maintain enough hope for them to not give up [27].

Optimism bias may also extend to technology. The belief that technology will be able to reverse or otherwise help humanity adapt to the adverse consequences of climate change may negatively affect climate change risk assessments by providing individuals with unrealistic expectations regarding the extent that future technology can compensate for current high carbon habits. Technological advances were one of the reasons that society has yet to reach the limit to industrialised growth that was predicted in the 1970s due to resource shortages [28]. A poll of 1534 American adults conducted by the Pew Research Center in 2016 found that 55% of respondents said they thought new technology will probably, or definitely, have solved most of the problems caused by global climate change in the next 50 years [29]. Expecting technology to solve climate change-related problems could reduce the incentive for individuals to change their carbon emission behaviours. In the U.K., technological optimism has been identified as a barrier to engaging in climate change mitigation [30]. That being said, the Pew Research Center reported that 67% of Americans stated major changes to lifestyle were necessary to address climate change [29]. This suggests that the majority of individuals do understand that they cannot solely rely on technological advances to mitigate climate change and that they will be required to change their behaviour in order to meet mitigation targets.

Relying on future technology to offset climate change has long been recognised as a gamble. First, the technology could fail to be invented in time to effectively reduce carbon emissions. Second, the technology may not be able to meet its targets [31]. Third, the technology could have detrimental side effects [31]. Behavioural research has shown that when gambling, people change their conceptions of probability depending on whether or not the payoff will occur in the near or distant future [32, 33]. For example, American university students were more likely to make riskier bets if the payoff was in the distant future compared to the near future [32]. This was true even when the gambles included loss [33]. Shelley explained that while people tend to discount both distant future gains and losses, compared to near future gains and losses they disregard distant losses at a greater rate [33]. This could help explain why people are willing to gamble on technology being able to help solve climate change issues even though it is risky.

Anderson criticised the IPCC’s climate change scenarios for the optimistic assumption that new climate change technology (such as geoengineering and other negative emissions technologies) will be available in the future [34]. Geoengineering refers to technology that can intentionally manipulate the earth’s climate [35], whereas negative emissions technologies intentionally remove carbon dioxide from the atmosphere [36]. Some examples of proposed geoengineered solutions to reduce atmospheric carbon include releasing aerosols into the atmosphere, space-based reflective shields, and carbon capturing and carbon sequestering [31]. Including technology that may not exist in future scenarios could give policymakers an unrealistic expectation of how much mitigation is required to keep earth habitable, and thus, they may develop response plans based on inferior information. Similarly, it has been suggested that technological myths may have been used by the aviation industry to reduce the perceived need for consumer action or policy responses [37]. By highlighting new or developing technology the aviation industry can give the public the false impression that they are close to creating low carbon forms of aeromobility even though these technologies may never eventuate or prove infeasible [37].

#### Travel in the year 2050

Climate change affects many things. This research focused specifically on visualizations of future travel for two reasons. First, the transport sector is a significant contributor to global emissions producing an estimated 23% of all greenhouse gas emissions in 2010 [38]. This is expected to increase particularly within the aviation industry despite industry promises that they will reduce their emissions [37]. Second, areas within the tourism industry are being affected by climate change (for example ski fields and coral reefs) [39]. Thus, depending on how travel is defined, it could both cause and be affected by climate change.

Spence, Poortinga and Pidgeon measured temporal distance by asking participants when they expected climate change to affect Great Britain [9]. This study took a slightly different approach to temporal distance by asking respondents about a specific time point in the future–the year 2050. The year 2050 was selected for two reasons: 1) it is not necessarily beyond participants’ lifetime thereby potentially increasing its personal relevance [40], and 2) it is a symbolic year often used by the IPCC when discussing climate change [1].

## Materials and methods

### Survey instrument

An online survey was used to collect both quantitative and qualitative data. Participants’ ability to visualize the future was measured using a multiple-choice question asking participants to self-report how clearly they could imagine the year 2050. This question was inspired by a question developed by Tonn, Hemrick, and Conrad which asks how clearly participants could imagine the future over a range of timeframes from 1 year to 100 years in the future [19]. We could have primed participants by first asking them questions that would help them envision specific aspects of the future; however, since we suspected that people do not normally spend much time thinking about the distant future we were more interested in their instinctive self-assessment of their ability to visualize the year 2050. For this reason, the question regarding participant’s self-reported ability to imagine the year 2050 was asked immediately following the demographics questions without any leading questions. Likert scale questions were used to assess participants’ optimism and attitudes towards technology and environmental beliefs. Since survey length can affect response rate [41], we limited the number of questions from established self-reported scales. As a measure of optimism, two statements (*I rarely count on good things happening to me* and *I am always optimistic about my future*) were used from The Live Orientation Test Revised [42]. To assess technological optimism, two statements (*Advancing technology provides us with hope for the future* and *Future resource shortages will be solved by technology)* were used from the Dominant Social Paradigm [43].

Concern towards climate change was measured in two ways. First participants were asked to rank seven global threats including climate change. These threats were taken from Lowe et al. [44], although it should be noted an eighth threat of poverty was inadvertently dropped from our study due to a human copy error. Next, participants answered a multiple-choice question asking them to select their level of concern regarding climate change. Environmental and societal beliefs were measured by two statements from The New Environmental Paradigm (*Humans have the right to modify the natural environment to suit their needs* and *There are limits to growth beyond which our industrialized society cannot expand*) [45]. Demographic information was collected through multiple choice questions. Lastly an open-ended question was used to explore participants’ perceptions of future travel. It did not define travel, providing freedom for participants to interpret the questions.

### Data collection and analysis

Data (S1 Table) were collected through an online survey (S1 File) hosted by SurveyMoneky^TM^ on 19–20 October, 2016. Participants were recruited through SurveyMonkey^TM^ with instructions to recruit at least 1000 participants with an even gender split. The survey required participants to give their informed consent before proceeding to the questionnaire. To be eligible, participants needed to be at least 18 years old and living in the USA. A total of 1143 people completed the survey. Responses were eliminated if participants did not answer the question which investigated attitudes towards optimism, technology and environmental beliefs (n = 72). Therefore, the total number of responses analysed was 1071. The study was approved by the Human Research Ethics Committee at the University of Otago (Ref: D15/393).

All quantitative data were analysed using SPSS^TM^ version 24. Descriptive statistics were used to determine how participants answered the multiple choice and Likert scale questions while relationships between variables were identified through Spearman’s correlations. We used hierarchical multiple regressions to test our models. Participants were asked to rank seven global threats, with one being the threat they were most concerned about and seven as being the threat they were least concerned about. Not all participants ranked all seven threats. It was assumed that unranked global threats represented a lack of concern. In an attempt to capture this lack of concern without skewing the rankings, these values were flipped during the analysis so that the threat that was most concerning was given a value of seven and the least concerning threat was given a value of one. Similarly, although both positive and negative statements were asked, all statements were converted into a positive framing for analysing and presenting the data.

Thematic coding was used to identify patterns within the open-ended question asking participants to describe their perceptions of future travel [46]. Representative words or phrases (ie code) were assigned to each response [47]. To increase validity and replicability of the results, a coding manual was designed (S2 Table) and intercoder reliability was calculated. The lowest percent agreement between the two coders was 92% and the lowest Krippendorff’s α was 0.732 suggesting there was a high level of consensus and replicability regarding the results [48]. A single response could contain multiple codes; for example: the quote “High speed rail. Hopefully more mass transit, fewer cars. More safe walking/biking paths” would be coded under ***shift*** because it refers to shifting to alternate modes of transportation (mass transport, walking and biking) as well as ***improve*** as it is assumed that references to faster trains or high-speed rail are referencing new technology such as Maglev trains (S2 Table).

### Participant demographics

Of the 1071 respondents, 52% (n = 557) identified as female, 46% (n = 491) identified as male and the remaining 2% identified as other (n = 8), preferred not to say (n = 9) or did not answer the questions (n = 6; S1 Fig). All age groups had at least 150 participants (S1 Fig). Participants aged 60 and over made up the largest age group (25%) while the least represented age group was between the ages of 40–49 (16%). Participants came from a wide variety of educational backgrounds (S1 Fig). An undergraduate degree was the highest qualification obtained by 27% of respondents. High school diplomas (20%), community college/technical college degrees (19%,) and master’s degrees (18%) were also common. Based on 2016 data from the US Census Bureau, the proportion of male and females in our study, as well as the age distribution of our participants were representative of the national data [49]; however, participants in this study tended to have attained higher levels of education than those reported in a 2016 report by the US Census Bureau [50]. Studies comparing participants recruited via SurveyMonkey have found responses to be within 10% comparison of traditional market research companies [51] and within a discrepancy of 5 to 10% between demographics of online respondents and their US distribution [52].

## Results and discussion

We tested the hypothesis that participants who had difficulty visualizing the distant future and a high level of future optimism would show lower levels of concern about climate change (Fig 1). A hierarchical multiple regression analysis revealed that neither participants’ self-reported ability to visualize the year 2050 nor participants future optimism were able to significantly explain the variance in individuals’ levels of concern about climate change (Table 1, Model 1). These results were contrary to our expectations.

**Table 1:** Hierarchical multiple regression analysis of the predictors of climate change concern.

	Model 1	Model 2	Model 3	Model 4	Model 5
Predictor Variables	β	Β	β	β	β
**Level 1**					
Ability to visualise the future	0.035	0.034	0.025	0.024	0.033
**Level 2**					
Future Optimism	0.058				
*Personal Optimism*		0.00	0.015	0.018	0.008
*Technological Optimism*		0.078*	0.156***	0.158***	0.158***
**Level 3**					
Environmental Beliefs			0.321***	0.321***	0.299***
**Level 4**					
Age				-0.038	-0.038
**Level 5**					
Gender					-0.133*
**Adjusted R**^**2**^	0.003	0.005	0.100	0.100	0.116
**ΔR**^**2**^	0.003	0.006	0.096	0.001	0.017

*p<0.05

**p<0.01

***p<0.001

**Fig 2:**
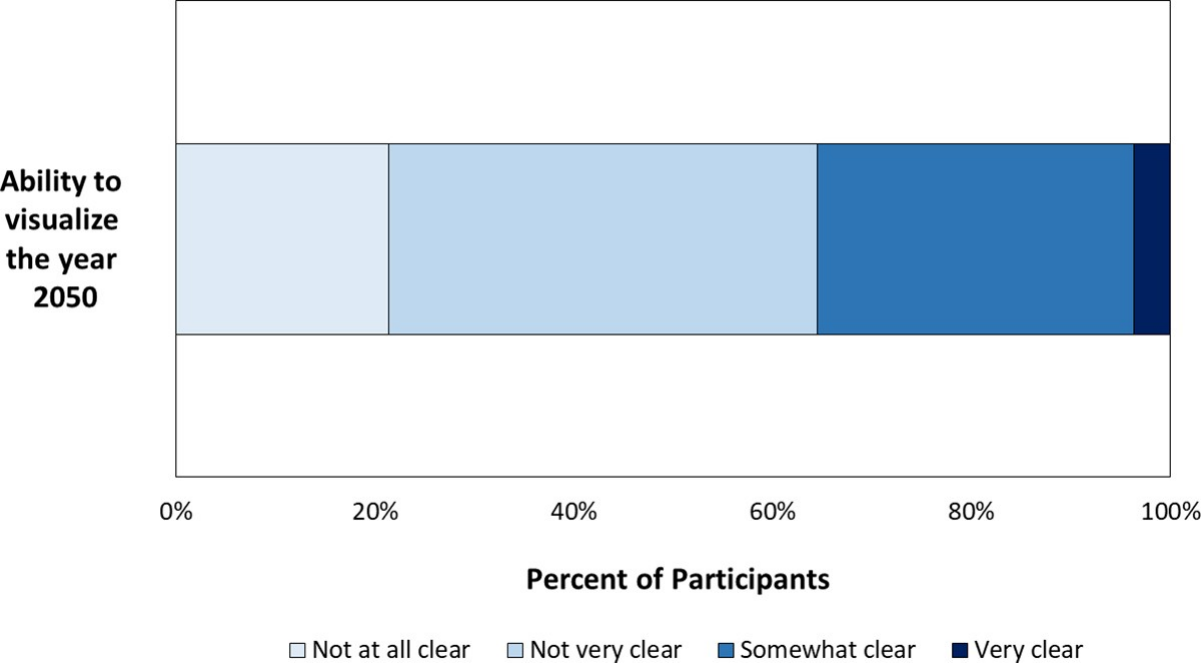
Participants’ self-reported ability to visualize the year 2050.

Consistent with findings of Tonn, Hemrick, and Conrad [19], the majority (64%) of our participants said they had trouble visualising the year 2050. Of the 1067 respondents who answered the question, 21% (n = 228) stated that the year 2050 was ‘not at all clear’ and 43% (n = 460) stated it was ‘not very’ clear (Fig 2). We had predicted that because the consequences of climate change are often discussed as future events [1], people who find it difficult to visualise the distant future may be less concerned about climate change. Our results suggest that this is not true.

**Fig 3:**
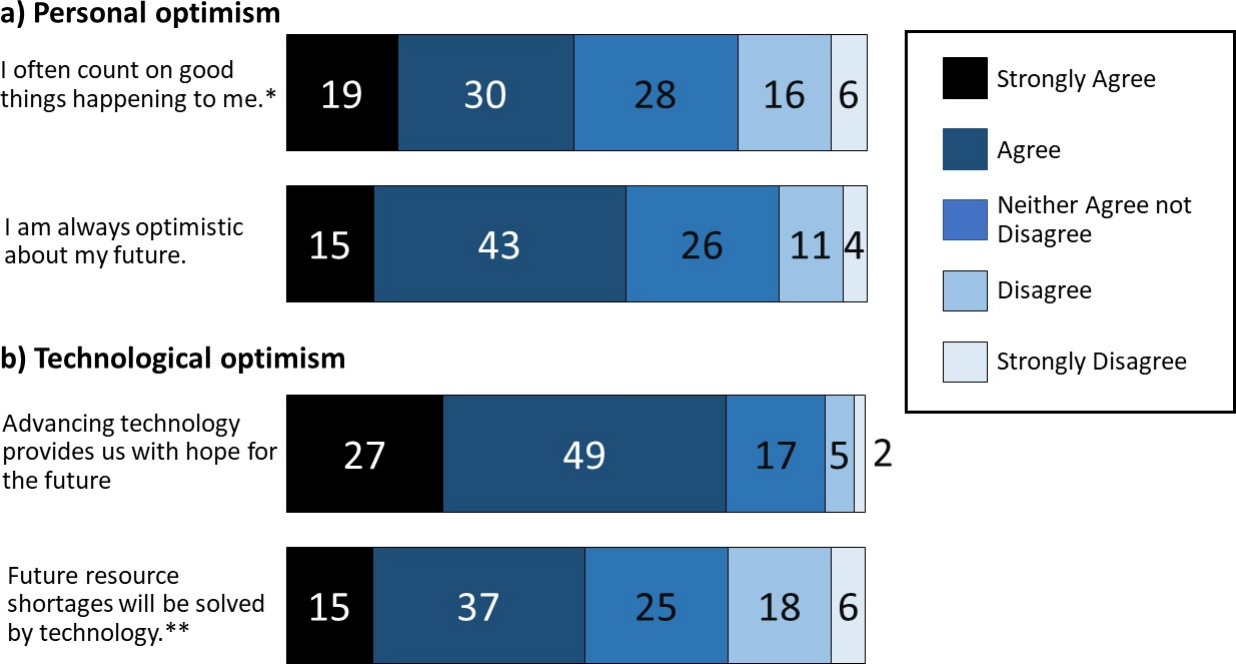
Percentage agreement on statements regarding participants’ personal optimism (a) and technological optimism (b). (N = 1068–1071) *original wording: I rarely count on good things happening to me. ** original wording: Future resource shortages will not be solved by technology.

We also predicted that participants would be optimistic about the future and that this, in turn, would make them more optimistic about the future effects of climate change. As expected, our respondents tended to be optimistic about their personal futures (Fig 3A). There was also large optimism surrounding the role of technology (Fig 3B). In fact, 76% of participants either ‘agreed’ or ‘strongly agreed’ with the statement that *advancing technology provides us with hope for the future* (Fig 3B). Interestingly, participants who were more likely to agree that future technology provides hope for the future were also more concerned about climate change (Spearman’s r(1035) = 0.123, p < 0.001).

Since there was a correlation between the statement *future technology provides hope for the future* and participants’ level of concern about climate change, we re-tested our hypothesis this time separating personal optimism and technological optimism (Model 2). We found that, while technological optimism was identified as a significant predictor of levels of climate change concern (Table 1), Model 2 still only explained < 1% of the variance. The results from Model 1 and Model 2 suggest that our original hypothesis that self-reported ability to visualize the distant future and future optimism would be good predictors of climate change risk perception is not valid.

One explanation for the rejection of our hypothesis could be that our initial assumption that Americans were not concerned about climate change was incorrect. When this research was conceived, the published literature suggested that Americans held moderate levels of climate change concern [53–55]. We found that 44% (n = 452) of our respondents stated they were ‘very concerned’ about climate change, 30% (n = 312) said they were ‘somewhat concerned’, 14% (n = 145) said they were ‘not too concerned’ and 9% (n = 88) were ‘not at all concerned.’ Only 4% of respondents (n = 40) said that they did not believe that climate change exists. The respondents also ranked climate change as the second most concerning issue behind terrorism (Table 2). Unsurprisingly, people who were more concerned about climate change were more likely to rank climate change as their top threat (r(1004) = 0.727, p < 0.001). The fact that climate change elicited a high level of concern and was ranked as a top threat suggests that climate change was perceived to be a serious threat to our American participants.

**Table 2:** Seven global threats ranked by level of concern, with higher rank indicating greater concern.

Global Threat	Overall Rank	Mean Rank	N
Terrorism	1	5.2	1020
Climate Change	2	4.8	1007
Loss of biodiversity	3	4.1	999
Radioactive waste	4	4.0	997
Nuclear power	5	3.9	1004
Genetic modification	6	3.5	996
AIDS	7	2.6	997

Another factor to consider is that we relied on participants’ self-reported ability to visualize the future. In addition to getting participants to describe future travel, this paper would have been stronger if we had asked participants to describe what they believed would be the consequences of climate change affecting the year 2050. We may have then been able to use these descriptions as another way of measuring ability to visualize the future. However, since many of our responses regarding future travel were brief this may be more appropriate in an interview setting where there is more time and the interview can tease out different ideas. Future studies could attempt to create a more objective measure to assess participants’ ability to visualize the distant future. Future research could also see if there is a difference in how people think of climate change before and after they are asked to describe how they envision the world in the future.

### Other predictors of climate change risk perception

The previous sections revealed that the original models (Model 1 and Model 2) should be rejected as they were not good predictors of participants’ levels of climate change concern. For Model 3, we tested whether environmental beliefs were a better predictor of levels of climate change concern. Since climate change threatens natural environments [1] it makes sense to test whether individuals’ environmental beliefs are related to their perceptions of climate change. A New Zealand study found environmental values to be positively correlated to climate change risk perception [56], and a U.K. study reported that next to direct experience with air pollution, environmental beliefs were the biggest predictor of engaging in climate mitigating actions such as taking public transport, walking or cycling [57]. Unfortunately, Whitmarsh did not present the data supporting this claim, making it difficult to verify [57]. Additionally, an Australian study found that beliefs regarding the ‘elasticity’ or ‘ductility” of the environment were related to whether participants said climate change is anthropomorphic in nature [58].

**Fig 4:**
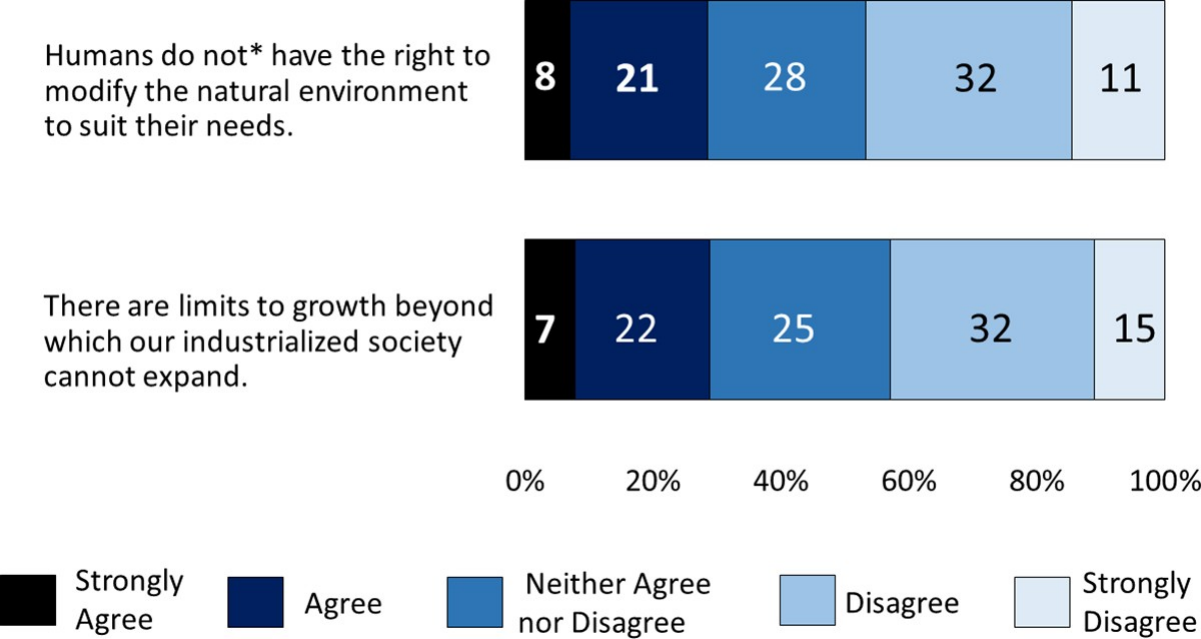
Percentage agreement with statements regarding participants’ environmental beliefs. (N = 1068–1071) *original wording: Humans have the right to modify the natural environment to suit their needs.

Our participants reported low levels of environmental concern with only 29% agreeing that *there are limits to growth beyond which our industrialised society cannot expand*. Similarly, only 29% of participants agreed that *humans do not have the right to modify the natural environment to suit their needs* (n = 309 and n = 304; Fig 4). When we added environmental beliefs to Model 3 the hierarchical regression found environmental beliefs to be a significant predictor of climate change concern, explaining 10% of the variance (Table 1, Model 3). These results demonstrate that environmental beliefs are a better predictor of climate change risk perception.

**Fig 5:**
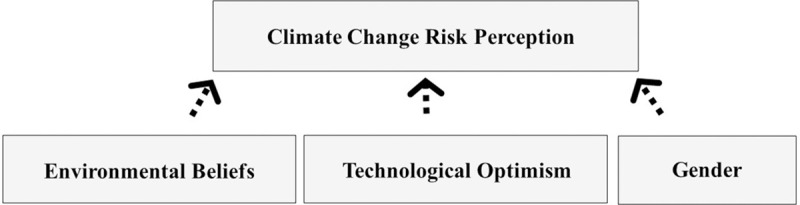
New model.

Lastly, we tested whether adding age (Model 4) and gender (Model 5) would increase the predictive power of the model. For example, older people may be less concerned about climate change since they might not expect to live to see the consequences of climate change [40]. Additionally, an American study found women tended to be more concerned about climate change [59]. Of the two variables, only gender was a significant predictor of participants’ levels of climate change concern (Table 1), with women being more concerned about climate change than men. Age as a factor affecting climate change concern was not supported by our data (Table 1). Overall, we found that technological optimism, environmental beliefs and gender were the best predictors of a person’s level of climate change concern (Fig 5). Self-reported ability to visualize the future and personal optimism were not major influencers in individuals’ judgments regarding climate change risk.

We did not ask participants about their political affiliations. This was an oversight as studies have shown that people who identify as Democrats are more likely to be concerned about climate change than people who identify as Republican [60, 61]. A Gallup poll from December 2019 found that Democrats and Independents were more likely to state climate change is an extremely important election issue for them than Republicans whereas terrorism was an important consideration for all three political groups [62]. Therefore, future studies interested in examining factors that could contribute to climate change risk judgement should include political views, values and worldviews. That being said, a study investigating Americans’ perceptions of what they think future energy systems will look like in the year 2050 found little difference between liberals and conservatives regarding what they expect to be the main energy sources [63]. This could suggest that Democrats and Republicans may have similar expectations of how climate change will affect the future; however, this would need to be further explored in future studies.

### Do participants visualize travel in 2050 as becoming more environmentally sustainable?

The previous section revealed that 44% of participants were very concerned about climate change and another 30% were somewhat concerned. We wanted to see if this concern translated into an expectation of a future with lower carbon travel. Of our participants, 29% (n = 310) described travel in the year 2050 as becoming more environmentally sustainable. For comparison other popular depictions of future travel included the idea that it would be faster (30%, n = 323) and that there would be driverless vehicles/more automation (22%, n = 231). A Spearman’s correlation found participants’ levels of concern about climate change concern was weakly correlated to whether they discussed future travel as becoming more environmentally sustainable (r(1049) = 0.142 p < 0.001) with people who were more concerned about climate change being more likely to describe future travel as being more environmental sustainable in 2050. These results suggest that being concerned about climate change might influence some people’s expectations regarding future travel.

There are two reasons why it would be beneficial to have people expecting a low carbon mobility future. First, if individuals think of low carbon mobility as inevitable they may be less resistant to change. Englehardt et al. noted that perceived inevitability is linked to public acceptance [64]. Perceived inevitability was one reason given by Americans for why they changed their views regarding same-sex marriage [65]. Herek suggested that attitude change regarding inevitability could result from trying to fit into changing social norms [65]. If people start perceiving low carbon mobility systems as being an inevitable change due to a shift in social values their attitudes regarding high and low carbon systems might change so that they fit in with social norms. The roles of values and social norms in determining attitudes and behaviours is widely described [66, 67]. Our results are consistent with the role of environmental values and social norms in contributing to increased sustainability of travel behaviours.

A second reason it would be beneficial to have people expecting a low carbon mobility future is that if individuals expect future mobility to transition to low carbon systems they may adopt low carbon technology sooner. For example, if an individual expects mobility systems to switch from petrol to electricity, they may be more inclined to purchase an electric vehicle the next time they buy a car. Studies profiling early adopters of electric vehicles have not included perceptions of future mobility systems in their investigations, making it difficult to determine the extent to which perceived inevitability may influence purchase behaviours. However, studies have found environmental attitudes to be an important predictor of purchasing electric vehicles [68–70].

Of the factors we tested in our models, environmental beliefs were the best predictor of an individual’s level of climate change concern. Interestingly, 29% of our study’s participants agreed with the pro-environmental statements provided–the same percentage of participants that described future travel as becoming lower carbon. However, environmental beliefs were not correlated to whether participants discussed future travel as becoming more environmentally sustainable (Spearman’s, N = 1071, p = 0.148). This suggests that both values being 29% is a coincidence and not simply that participants with strong environmental values were the same people who expected future travel to become more environmentally sustainable.

Another predictor of climate change concern was technological optimism. The Avoid/Shift Improve framework argues that when transitioning to low carbon transportation we should be aiming to avoid travel (ie less physical travel) [71]. The next most effective option is to shift away from current high carbon modes of transportation (ie switch from driving personal vehicles to taking public transportation) and lastly we can improve current technology to utilize less carbon (ie use electric vehicles instead of gasoline powered vehicles) [71]. Looking at participants’ depictions of future travel through the Avoid/Shift/Improve transitional framework [71, 72], we found that technological improvements were often discussed when describing lower carbon travel options (Table 3). For example, participants suggested that vehicles would use different fuel sources such as electric, solar, or hydrogen. One participant explained, “I think all cars will be electric, or some other environmentally friendly form of fuel [Female, age 50–59].”

**Table 3:** Percentage category frequency of participants’ expectations regarding environmentally sustainable travel in the year 2050.

Sub-theme	N	Percent
Improve	161	15
Shift	150	14
Avoid	46	4
Other	41	4

(N = 1071)

Even when participants discussed shifting to alternate modes of transportation (such as trains), technological improvements were often mentioned as an underlying reason for the switch. Participants talked about Maglev and bullet trains being able to travel faster than the trains that are currently in use in the United States and thus being more appealing.

California will have a bullet train aka high speed railroad from San Francisco to Los Angeles thus reducing need for intra-state air flights. Car manufacturers like Tesla pushing self driving car technology onto the market will redefine/shift luxury car market from cars w/leather seats to cars able to drive themselves, thus also increasing public transportation buses since cities no longer limited to finding certified commercial drivers [Male, age 30–39].

Similarly, avoiding travel could be aided by technological improvements that create virtual realities and virtual tours. A different participant wrote that they thought future travel would involve:

"Traveling" via virtual reality: Interactions with real people—designated staff at attractions, for example—who can lead real-time guided tours with people who are logged in from their physical location. [Gender unreported, age 40–49].

Overall, the written depictions provided by participants regarding their expectations of travel in the year 2050 revealed that while being concerned about climate change increased the likelihood that an individual would describe future travel as becoming more environmentally sustainable, low carbon travel was not likely the first thing people think about when discussing future travel. It also reinforced the idea that participants expect technology to play a large role in mitigating climate change.

### Where things could get complicated: Technological solutions and environmental beliefs

One of our most important results was that participants who were more concerned about climate change were more likely to agree that future technology provides hope for the future. This could be because participants who are more concerned about climate change may perceive technology as the best or most likely way to mitigate climate change. At the same time, participants in our study tended to disagree that humans have the right to modify the natural environment to suit their needs. The higher a participant’s level of climate change concern, the more likely they were to disagree with this statement. This poses an interesting question regarding what types of climate mitigating technologies may or may not be acceptable to participants. For example, the presence of high-speed train systems across the United States was frequently mentioned as a way that travel would change by the year 2050. Although high speed trains have the potential to make future travel more sustainable they would require new infrastructure, raising the question: would people find it acceptable to disrupt natural ecosystems in order to create systems that could help reduce carbon emission?

A more extreme solution would be geoengineering. A workshop looking at public perceptions of geoengineering in the UK found participants tended to view geoengineering as interfering with nature but were divided as to whether geoengineering was a harmful or necessary mitigation strategy [35]. The acceptability of geoengineering as a method of climate change mitigation may be dependent on personal values [73]. Results from the 2011 National Survey of American Public Opinion on Climate Change found that Americans who believed in climate change were more likely to agree that humans can use geoengineering to combat climate change, whereas those who did not believe in climate change were more likely to agree that geoengineering will do more harm than good [74]. Perhaps those who are more concerned about climate change reduce cognitive dissonance by justifying geoengineering as an acceptable or necessary modification of nature.

## Conclusion

Americans will have an enormous responsibility, and equally significant role to play in mitigating climate change. Since climate change risk perception may influence climate mitigating behaviours, we hypothesized that the ability to visualize the future and future optimism could influence climate change risk perception. Our data on Americans’ perceptions of climate change risk were collected a few weeks before the 2016 American Presidential election; thus, this research also provides a snapshot of public opinion at a critical time in climate change history.

We offered a framework that tried to indirectly test how the distant future could influence perceptions of climate change risk. We found that self-reported ability to visualize the distant future and personal optimism were not good predictors of levels of concern about climate change. However, we did find that technological optimism was correlated to climate change concern. Future research could explore the underpinning reasons for this relationship and whether it applies to all forms of climate mitigating technology or only certain proposals.

We found that environmental beliefs were a stronger predictor of climate change than the factors that we initially proposed. Future research interested in factors that affect climate change risk perception should continue to include environmental values. It will be beneficial for researchers to focus on how to convert a growing concern about climate change into actionable mitigation strategies. We believe that achieving a low carbon future will be easier if people associate the future with low carbon options. Future research should continue to explore methods of engaging people in climate mitigating behaviours.

## Supporting information

S1 File Online survey questions. This is a PDF of the online survey that participants were required to complete.
(PDF)

S1 Fig Participant demographics by gender (a), age (b) and highest level of education (c).
(TIF)

S1 Table Raw data collected from survey respondents. This is a table displaying the answers given by participants to the survey questions (to view survey questions refer to S1 File).
(XLSX)

S2 Table Coding categories. The data was coded using the following four rules: 1) If the theme is present mark 1. 2) The specific number of times a theme or sub-theme is mentioned within one quote ***is not*** recorded. Only the presence or absence of the theme or sub-theme. 3) Quotes can belong to multiple themes and sub-themes. 4) If an answer has conflicting views or multiple depictions of the future code for all.
(DOCX)

S3 Table Correlation matrix of the independent variables. (DOCX)
