# Supplementary material for: Climate change risk perception in the USA and alignment with sustainable travel behaviours
Source: PLoS One. 2021 Feb 3;16(2):e0244545. doi: 10.1371/journal.pone.0244545 (PMC7857622; doi:10.1371/journal.pone.0244545)
Supplement: S1 File — This is a PDF of the online survey that participants were required to complete. (PDF) [file pone.0244545.s001.pdf]

Traveling Toward 2050

Welcome to the survey Travelling Towards 2050

What is the aim of the project?

This is a survey carried out by researchers from the University of Otago investigating people's perceptions of travel in the year 2050.

What types of participants are being sought?

We are looking for participants over the age of 18.

What will participants be asked to do?

The survey should take less than 10 minutes to complete. Taking part in this survey is voluntary. No identifying data will be collected and you can withdraw at anytime without any disadvantage to yourself.

The data collected will be securely stored in a lockable drawer in a locked office in the Centre for Science Communication. Data obtained as a result of the research will be retained for at least 5 years in secure storage. The results of the project may be published and will be available in the University of Otago Library (Dunedin, New Zealand) but every attempt will be made to preserve your anonymity.

This study has been approved by the University of Otago Human Ethics Committee. However, if you have any concerns about the ethical conduct of the research you may contact the University of Otago Human Ethics Committee through the Human Ethics Committee Administrator (ph +64 3 479 8256). Any issues you raise will be treated in confidence and investigated and you will be informed of the outcome.

If you have any questions about this project, either now or in the future, feel free to contact either:

Jean Fletcher (fleje401@student.otago.ac.nz)
Prof Nancy Longnecker (nancy.longnecker@otago.ac.nz)
Centre for Science Communication, University of Otago
+(64) 3 479 7885

Thank you for your help!

* 1. **By selecting "yes" I agree to the following:**
i) That I am over the age of 18
ii) That I understand the information associated with this project.
iii) I am willing to participate in this project

- ◯ Yes

Traveling Toward 2050

Demographics

2. I am:

- ◯ 18-19
- ◯ 20-24
- ◯ 25-29
- ◯ 30-34
- ◯ 35-39
- ◯ 40-49
- ◯ 50-59
- ◯ 60+

3. I am:

- ◯ Male
- ◯ Female
- ◯ Other
- ◯ I'd rather not say

4. I am (nationality):

5. The highest level of education that I have completed is:

- ◯ High School Diploma
- ◯ Community College/ Technical College
- ◯ Undergraduate
- ◯ Postgraduate diploma/ certificate
- ◯ Master's Degree
- ◯ PhD
- ◯ Apprenticeship
- ◯ Other (please specify)

UNIVERSITY of OTAGO Te Whare Wānanga o Otāgo NEW ZEALAND

Traveling Toward 2050

Visualizating the Future

6. How clearly can you imagine the year 2050

Not at all clearly	Not very clearly	Somewhat clearly	Very clearly
◯	◯	◯	◯

7. Do you think travelling in 2050 will be any different than travelling in 2015?

- ◯ Yes
- ◯ No
- ◯ I don't know

UNIVERSITY of OTAGO Te Whare Wānanga o Otāgo NEW ZEALAND

Traveling Toward 2050

Visualizing the Future Continued

8. How do you think travelling in 2050 will be different than travelling in 2015?

UNIVERSITY of OTAGO
Te Whare Wānanga o Otāgo
NEW ZEALAND

Traveling Toward 2050

9. Indicate the extent to which you agree with the following statements.

	Strongly Disagree	Disagree	Neither Agree nor Disagree	Agree	Strongly Agree
I rarely count on good things happening to me.	○	○	○	○	○
Advancing technology provides us with hope for the future.	○	○	○	○	○
Business interests have more political power than individuals.	○	○	○	○	○
Humans have the right to modify the natural environment to suit their needs.	○	○	○	○	○
I am always optimistic about my future.	○	○	○	○	○
Future resource shortages will not be solved by technology.	○	○	○	○	○
There are limits to growth beyond which our industrialized society cannot expand.	○	○	○	○	○

UNIVERSITY of OTAGO
Te Whare Wānanga o Otāgo
NEW ZEALAND

Traveling Toward 2050

Risk

10. Rank in order of concern (*with 1 representing the issue you are most worried about*)

- AIDS
- Radioactive waste
- Loss of biodiversity
- Genetic modification
- Climate change
- Nuclear power
- Terrorism

11. I am:

- ○ Very concerned about climate change
- ○ Somewhat concerned about climate change
- ○ Not too concerned about climate change
- ○ Not at all concerned about climate change
- ○ Climate change does not exist

Traveling Toward 2050

12. If you have any other comments please provide them here.

Thank you so much for your participation.
