# Supplementary figures and images for: Climate change risk perception in the USA and alignment with sustainable travel behaviours
Source: PLoS One. 2021 Feb 3;16(2):e0244545. doi: 10.1371/journal.pone.0244545 (PMC7857622; doi:10.1371/journal.pone.0244545)

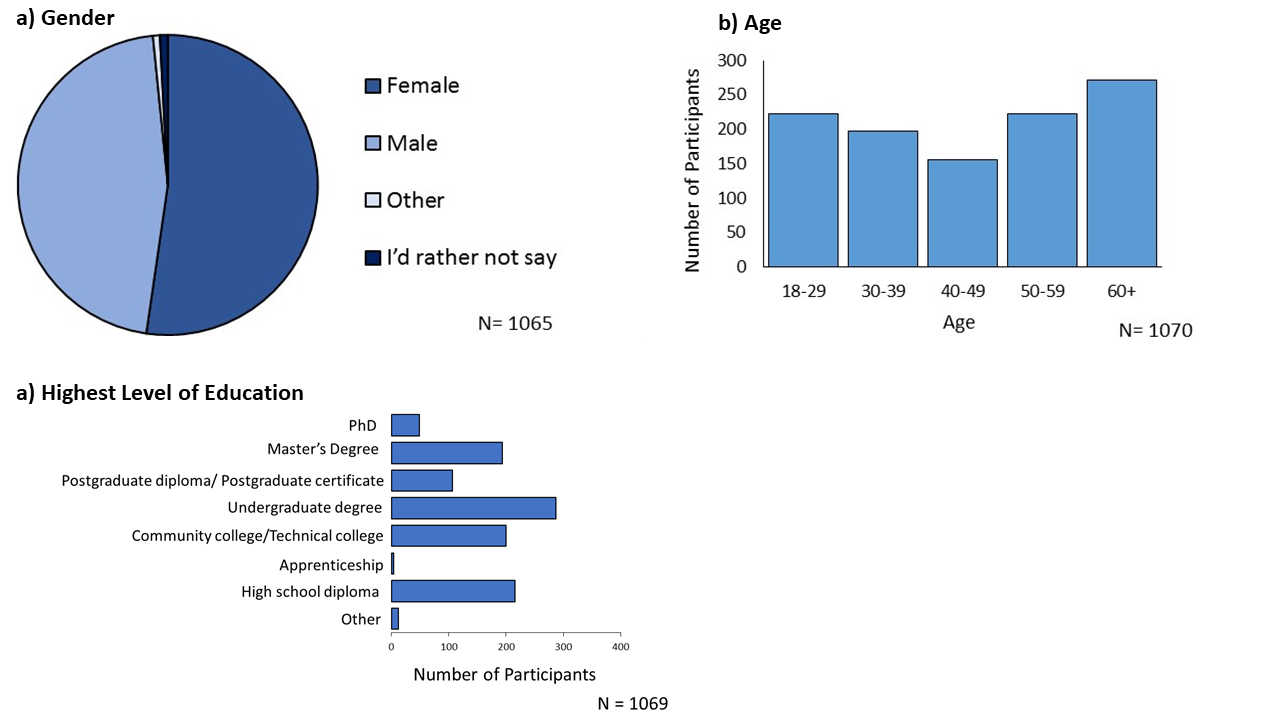

Supplement: S1 Fig — Participant demographics by gender (a), age (b) and highest level of education (c). (TIF) [file pone.0244545.s002.tif]
