# Supplementary material for: Climate change risk perception in the USA and alignment with sustainable travel behaviours
Source: PLoS One. 2021 Feb 3;16(2):e0244545. doi: 10.1371/journal.pone.0244545 (PMC7857622; doi:10.1371/journal.pone.0244545)
Supplement: S2 Table — The data was coded using the following four rules: 1) If the theme is present mark 1. 2) The specific number of times a theme or sub-theme is mentioned within one quote is not recorded. Only the presence or absence of the theme or sub-theme. 3) Quotes can belong to multiple themes and sub-themes. 4) If an answer has conflicting views or multiple depictions of the future code for all. (DOCX) [file pone.0244545.s004.docx]

**S2 Table.** **Coding categories.**

| **Theme** | **Sub theme** | **Description** | **Include** | **Exclude** | **Examples** |
| --- | --- | --- | --- | --- | --- |
| Future travel will become more environmentally sustainable | Avoid | - References to decreasing travel | - Less travel, - More virtual travel, - More telecommuting, - More local travel, - Travel is restricted | - Reference that could discourage travel (harder, more difficult, more expensive), - General references that say travel will change but do not specify in which direction | - “…international travel may be more restricted due to stricter immigration laws.” - “Virtual travel will become more common and more accepted.” |
|  | Shift | • References to an increased use of low carbon modes of travel (excluding electric vehicles) | - More public transport, - More trains/ highspeed trains/ Maglev/ light rail - More cycling, - More walking, - More horses, - Less individual car ownership/more car sharing | - Electric cars, - Electric vehicles - Alternate fuels | - “Greater use of rail transport, with high-speed bullet trains.” - “More walkable, bike-able cities.” - “Fewer personal/ privately owned autos. More shared vehicles.” |

| **Theme** | **Sub theme** | **Description** | **Include** | **Exclude** | **Examples** |
| --- | --- | --- | --- | --- | --- |
| Future travel will become more environmentally sustainable *(continued)* | Improve | • References to reducing carbon emissions through technological advancements | - Alternative fuel, - Electric/ Solar/ Hybrid/ Hydrogen vehicles - Increased fuel efficiency - Faster trains/ bullet trains/ hyperloop - Virtual reality | - Unspecified references to efficiency (because they could mean time or fuel), - Improvements to technology that is unrelated to low carbon mobility (eg. faster planes, self-driving vehicles) - Teleportation - No more fossil fuels (because you don’t know if this is due to changes in technology or mode of travel) | - “Fossil fuels will, hopefully, be replaced by clean energy so all types of vehicles will be electric, solar or maybe wind powered.” - “More bullet trains and monorail systems. High speed tubes.” |
|  | Other | - Any general catch-all phrase referring to travel becoming more sustainability, - References to less fossil fuels in use but without an explanation of the way that they will be reduced (ie improved fuel efficiency, more buses, less travelling etc). | - Eco-friendly, - Greener, - Less harmful to the environment, - More environmentally sustainable - Less pollution - No more fossil fuels | - Unspecified references to efficiency (because they could mean time or fuel), | - “Less pollution. More eco-friendly modes of transportation.” - “Fossil fuel, will not be needed.” |
