# Supplementary material for: Climate change risk perception in the USA and alignment with sustainable travel behaviours
Source: PLoS One. 2021 Feb 3;16(2):e0244545. doi: 10.1371/journal.pone.0244545 (PMC7857622; doi:10.1371/journal.pone.0244545)
Supplement: S3 Table — (DOCX) [file pone.0244545.s005.docx]

|  |  | How clearly can you imagine the year 2050 | I am always optimistic about my future. | I rarely count on good things to happening to me | Advancing technology provides us with hope for the future | Future resource shortages will not be solved by technology. | Humans have the right to modify the natural environment to suit their needs. | There are limits to growth beyond which our industrialized society cannot expand | Business interests have more political power than individuals. | Climate change concern |
| --- | --- | --- | --- | --- | --- | --- | --- | --- | --- | --- |
| How clearly can you imagine the year 2050 | Correlation Coefficient | 1.000 |  |  |  |  |  |  |  |  |
|  | Sig. (2-tailed) | NA |  |  |  |  |  |  |  |  |
|  | N | 1087 |  |  |  |  |  |  |  |  |
| I am always optimistic about my future. | Correlation Coefficient | 0.110^**^ | 1.000 |  |  |  |  |  |  |  |
|  | Sig. (2-tailed) | < 0.000 | NA |  |  |  |  |  |  |  |
|  | N | 1064 | 1068 |  |  |  |  |  |  |  |
| I rarely count on good things to happening to me | Correlation Coefficient | - 0.038 | - 0.396^**^ | 1.000 |  |  |  |  |  |  |
|  | Sig. (2-tailed) | 0.212 | < 0.000 | NA |  |  |  |  |  |  |
|  | N | 1064 | 1065 | 1068 |  |  |  |  |  |  |
| Advancing technology provides us with hope for the future | Correlation Coefficient | 0.104^**^ | 0.274^**^ | -0.142^**^ | 1.000 |  |  |  |  |  |
|  | Sig. (2-tailed) | 0.001 | < 0.000 | < 0.000 | NA |  |  |  |  |  |
|  | N | 1065 | 1066 | 1066 | 1069 |  |  |  |  |  |
| Future resource shortages will not be solved by technology. | Correlation Coefficient | - 0.050 | - 0.104^**^ | 0.138^**^ | -0.265^**^ | 1.000 |  |  |  |  |
|  | Sig. (2-tailed) | 0.099 | 0.001 | < 0.000 | < 0.000 | NA |  |  |  |  |
|  | N | 1066 | 1067 | 1067 | 1068 | 1070 |  |  |  |  |
| Humans have the right to modify the natural environment to suit their needs. | Correlation Coefficient | - 0.017 | 0.114^**^ | 0.058 | 0.079^*^ | - 0.023 | 1.000 |  |  |  |
|  | Sig. (2-tailed) | 0.574 | < 0.000 | 0.059 | 0.010 | 0.456 | NA |  |  |  |
|  | N | 1065 | 1066 | 1066 | 1067 | 1068 | 1069 |  |  |  |
| There are limits to growth beyond which our industrialized society cannot expand | Correlation Coefficient | - 0.024 | - 0.105^**^ | 0.081^**^ | - 0.126^**^ | 0.318^**^ | - 0.092^**^ | 1.000 |  |  |
|  | Sig. (2-tailed) | 0.425 | 0.001 | 0.008 | < 0.000 | < 0.000 | 0.003 | NA |  |  |
|  | N | 1066 | 1067 | 1067 | 1068 | 1069 | 1068 | 1070 |  |  |
| Business interests have more political power than individuals. | Correlation Coefficient | - 0.012 | - 0.082^**^ | 0.109^**^ | 0.034 | - 0.021 | - 0.114^**^ | .188^**^ | 1.000 |  |
|  | Sig. (2-tailed) | 0.701 | 0.007 | < 0.000 | 0.262 | 0.488 | < 0.000 | < 0.000 | NA |  |
|  | N | 1067 | 1068 | 1068 | 1069 | 1070 | 1069 | 1070 | 1071 |  |
| Climate change concern | Correlation Coefficient | 0.049 | - 0.021 | - 0.010 | 0.123^**^ | 0.003 | - 0.307^**^ | 0.092^**^ | 0.104^**^ | 1.000 |
|  | Sig. (2-tailed) | 0.115 | 0.501 | 0.759 | < 0.000 | 0.912 | < 0.000 | 0.003 | 0.001 | NA |
|  | N | 1033 | 1034 | 1034 | 1035 | 1037 | 1035 | 1037 | 1037 | 1037 |

*. Correlation is significant at the 0.05 level (2-tailed).

**. Correlation is significant at the 0.01 level (2-tailed)
